# Testosterone Is Associated with Erectile Dysfunction: A Cross-Sectional Study in Chinese Men

**DOI:** 10.1371/journal.pone.0039234

**Published:** 2012-06-21

**Authors:** Ming Liao, Xianghua Huang, Yong Gao, Aihua Tan, Zheng Lu, Chunlei Wu, Youjie Zhang, Xiaobo Yang, Haiying Zhang, Xue Qin, Zengnan Mo

**Affiliations:** 1 Institute of Urology and Nephrology, First Affiliated Hospital of Guangxi Medical University, Nanning, Guangxi Zhuang Autonomous Region, People’s Republic of China; 2 Center for Genomic and Personalized Medicine, Guangxi Medical University, Nanning, Guangxi Zhuang Autonomous Region, People’s Republic of China; 3 Center for Metabolic Disease and Diabetes, First Affiliated Hospital of Guangxi Medical University, Nanning, Guangxi Zhuang Autonomous Region, People’s Republic of China; 4 Department of Occupational Health and Environmental Health, School of Public Health of Guangxi Medical University, Nanning, Guangxi Zhuang Autonomous Region, People’s Republic of China; 5 Department of Clinical Laboratory, First Affiliated Hospital of Guangxi Medical University, Nanning, Guangxi Zhuang Autonomous Region, People’s Republic of China; 6 Fudan-VARI Center for Genetic Epidemiology, School of Life Science, Fudan University, Shanghai, People’s Republic of China; 7 Urology Department, Guigang People’s Hospital, Guigang, Guangxi Zhuang Autonomous Region, People’s Republic of China; Roswell Park Cancer Institute, United States of America

## Abstract

**Background:**

Testosterone is essential for the regulation of erectile physiology, but the relationship between low testosterone and erectile dysfunction (ED) has not been firmly established.

**Purpose:**

To examine the association between serum total, free and bio-available testosterone and ED in a population-based sample.

**Methods:**

A consecutive series of 1776 men aged 20–77 participated in the routine physical examination from September 2009 to December 2009 in Guangxi, China. ED was assessed using the five-item International Index of Erectile Function (IIEF-5) questionnaire. Total testosterone (TT), sex hormone binding globulin (SHBG) and other biochemical profiles were measured. Free testosterone (FT) and bio-available testosterone (BT) were calculated based on Vermeulen’s formula. Data were collected with regard to smoking, alcoholic drinking, physical activity and metabolic syndrome.

**Results:**

The prevalence of ED (IIEF-5<22) was 47.6%. Men with ED were significantly older, and more prone to smoke cigarettes (≥20 cigarettes/day) or drink alcohol (≥3 drinks/week), and more likely to have elevated blood pressure (P = 0.036) or hyperglycemia (P<0.001) compared with those without ED. The significant increase in SHBG with age was parallel to its increase with increasing severity of ED (P<0.001). The obscure increase in TT across the ED status was detected without significance (P = 0.418), but TT was positively associated with ED after adjustment for age [odds ratio (OR)  = 1.02, 95% CI (confidence internal): 1.00–1.04]. FT and BT were inversely associated with ED (OR = 0.14, 95%CI: 0.06–0.33; OR = 0.92 (95%CI: 0.89–0.96, respectively) in the univariate analysis, and this inverse association appeared to be independent of smoking status, alcoholic drinking, physical activity, hyper-triglyceridemia and hyperglycemia.

**Conclusions:**

FT and BT are inversely related to worsening ED, whereas the positive association between TT and ED is most likely due to the increase in SHBG.

## Introduction

Erectile dysfunction (ED) is a highly prevalent disorder among men all around the world [1]–[3], and possibly related to the rise in diabetes and vascular diseases [4]. Its incidence increases with age, and the ageing process in men is accompanied by a progressive decline in serum testosterone levels. Although testosterone deficiency is often found in patients presenting with ED alone, it is commonly not the principal cause [5]. Nevertheless, testosterone is increasingly considered in the clinical setting to treat ED [6], especially in those patients unresponsive to phosphodiesterase type 5 inhibitors, and often results in an improvement in sexual function [7]. Although there is some preliminary animal experimental evidence that testosterone is essential for the regulation of erectile physiology by multiple mechanisms [8], the causal relationship between low testosterone and ED has not been firmly established [5]. It is, therefore, important to further investigate the relationships between testosterone and erectile function, especially in a general population without the substantial biases inherent in patient samples.

Previously in a sample of Korean men with lower urinary tract symptoms [9], free testosterone (FT) was correlated with erectile function, consistent with the later study [10], but total testosterone (TT) was not correlated with any of the five domains of the international Index of Erectile Function (IIEF). In terms of other previous surveys, neither correlation between TT and ED risk nor with ED severity was demonstrated in studies of Brazil [11], [12], Turkey [13], [14] and Italy [15], though low TT was associated with sexual dysfunction more often in the oldest subjects [16]. With respect to bio-available testosterone (BT), it was correlated well with the erectile function assessed by IIEF-5 score in the sample of 130 outpatients from Japan [17]. And it was reported in the Olmsted County study, the age-related decline in sexual function was due to age-related declines in levels of BT rather than TT levels [18]. It is only fairly recently that testosterone threshold for the relationship between TT and ED has been found in European Male Ageing Study (EMAS) [5]. We conclude that the frustration to clarify the relationship between testosterone and ED in previous studies is probably due to the different provenances of studied population or the underpowered sample size. Moreover, to best of our knowledge, unhealthy lifestyles such as cigarette smoking, alcoholic drinking and physical activity [19], as well as the metabolic syndrome consisting of a myriad of abnormalities including central obesity, glucose intolerance, dyslipidemia, and hypertension [20] have been associated with ED, but few studies considered these factors. It is, therefore, in order to further evaluate the relationship between testosterone and ED with the consideration of these confounders of ED, that we conducted this cross-sectional study in a large series of Chinese men from general population.

**Figure 1:**
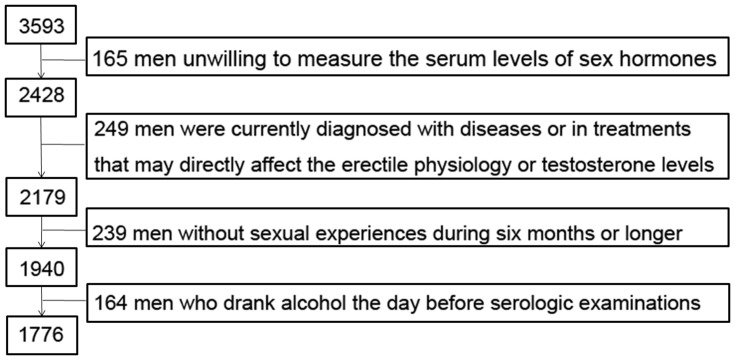
Flow chart of recruitment in the present study.

## Materials and Methods

### Study Population

Our analyses are based on the Fangchenggang Area Male Health and Examination Survey (FAMHES), which was designed to investigate the effects of environmental and genetic factors and their interaction with the development of age-related chronic diseases [21]–[23]. Briefly, the FAMHES was a population-based study conducted among non-institutionalized Chinese men aging from 17 to 88 years old in Guangxi, China. A comprehensive demographic and health survey was conducted among a consecutive series of 4303 men participating in the routine physical examination at the Medical Centre in Fangchenggang First People’s Hospital from September 2009 to December 2009. A total of 3,593 people completed the data collection interviews. There were no significant differences between these people and those who did not complete the interviews. The response rate was 83.5% [23], and all the participants provided written informed consents. The survey received the approval from ethics committee in Guangxi Medical University.

In the current cross-sectional study, participants were excluded based on the following criteria: (1) currently diagnosed with myocardial infarction, congestive heart failure, stroke, hyperthyroidism, rheumatoid arthritis, acquired immune deficiency syndrome and any kind of cancer, or with a history of pelvic trauma/surgery or suffering significant urinary tract infection; (2) in current treatment with herbal remedies, or with medication including psychotropic drugs, non-steroidal anti-inflammatory drugs, antibiotics, spironolactone, cimetidine, glucocorticoids or other steroidal drugs which might affect the testosterone level or drugs with effect on erectile physiology, such as dopamine-antagonists, diuretics and so on; (3) men without regular sexual experiences during six months preceding completion of the International Index of Erectile Function (IIEF-5) questionnaire or men with absence of sex hormones measurements; (4) who drank beer, wine or hard liquor the day before serologic examination. A flow chart indicating the process of enrolling was showed in Figure 1. We finally enrolled 1,776 men aged 20–77 years in the present study.

**Table 1:** Characteristics of the analysis sample at baseline in FAMHES*.

	**Erectile Dysfunction**		
	**Yes**	**No**	**P**
Number	845	931	
Age† - year	40 (31–47)	35(28–40)	<0.001
Race Han - %	760 (89.9)	820 (88.1)	0.72
TT † - nmol/l	20.5±1.4	20.0±1.4	0.212
FT †- nmol/l	0.39±0.1	0.41±0.1	0.006
BT † - nmol/l	9.3±1.3	9.7±1.3	0.007
SHBG † -nmol/l	38.9±1.36	34.9±1.6	<0.001
Cigarette smoking - %			0.05
Never smoker	388 (45.9)	422 (45.3)	
Former smoker	44 (5.2)	27 (2.9)	
<20 cigarettes per day	178 (21.1)	225 (24.2)	
≥20 cigarettes per day	235 (27.8)	257 (27.6)	
Alcoholic drinking - %			0.022
Never drinker	134(15.9)	107 (11.9)	
<3 drinks per week	567 (67.1)	668 (74.1)	
≥3 drinks per week	144 (17.0)	156 (17.3)	
Physical activities - %			0.922
<2 hours per week	630 (74.6)	696 (74.8)	
≥2 hours per week	215 (25.4)	235 (25.2)	
Metabolic Syndrome - %	104 (12.4)	97 (10.5)	0.207
Hypertriglyceridemia	232 (27.5)	271 (29.2)	0.407
Hyperglycemia	248 (29.3)	199 (21.4)	<0.001
Elevated BP	189 (22.4)	171 (18.4)	0.036
Low HDL-C	57 (6.7)	74 (8.0)	0.32
Central obesity	148 (17.7)	167(18.0)	0.855

† Age was present as median values (25–75th percentile); sex hormones including TT, BT and SHBG were logarithmically transformed in the following analysis and reported as back-transformed arithmetic mean values ± SD (standard deviation).

* FAMHES =  the Fangchenggang Area Male Health and Examination Survey, TT = total testosterone, FT = free testosterone, BT = bioavailable testosterone, SHBG = sex hormone-binding globulin, BP = blood pressure, HDL-C = high-density lipoprotein cholesterol. TT and FT values divide by 3.467 can be converted to ng/ml.

**Figure 2:**
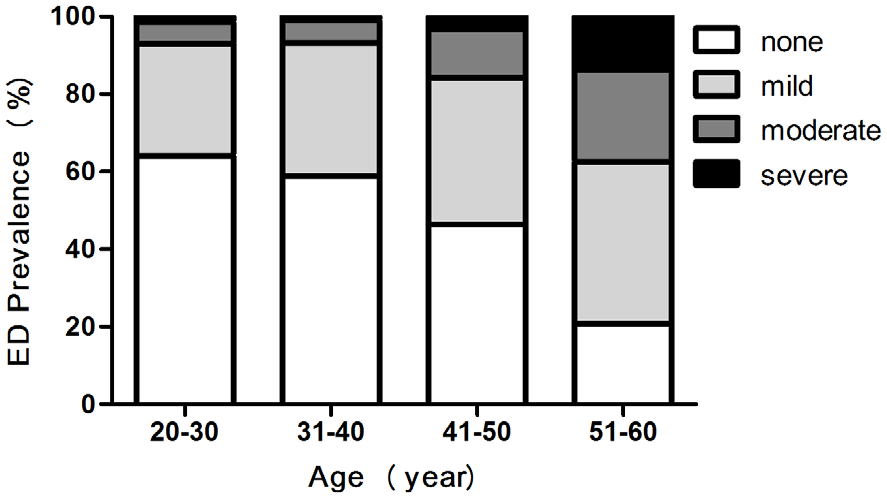
Prevalence of ED increases with age.

### Data Collection

ED was defined using the IIEF-5, a self-administered and validated instrument widely used in both clinical and epidemiologic studies [24]. The five items assess erection confidence, erection firmness, maintenance ability, maintenance frequency, and satisfaction. Each item is scored on a 5-point ordinal scale where lower values represent poorer sexual function. The IIEF-5 score ranges between 5 and 25 with lower scores indicating increased severity of ED. ED status was classified into five categories as none (IIEF-5 score 22–25), mild (17–21), moderate (12–16), and severe (5–11). Additionally, ED was defined as a dichotomous variable using a cut-off point of IIEF-5<22 (mild, moderate, and severe). This approach is similar to the definition of ED used in the Boston Area Community Health (BACH) Survey [25].

A complete physical examination including the measurement of waist circumference and blood pressure (BP) was performed on each subject. Waist circumference was measured at the midpoint between the inferior costal margin and the superior border of iliac crest on midaxillary line. BP was measured twice after resting for more than 15 min, with the mercury sphygmomanometer by well-trained nurses, and the average values were taken. Metabolic syndrome was defined based upon the updated report of National Cholesterol Education Program Adult Treatment Panel III (NCEP ATPIII criteria) for Asian Americans as having three or more of the following components: (1) waist circumference at least 90 cm, (2) triglycerides at least 1.7 mmol/L, (3) high-density lipoprotein cholesterol (HDL-C) less than 1.03 mmol/L, (4) BP at least 130/85 mm Hg, (5)fasting glucose at least 5.6 mmol/l [26]. Smoking status was defined as never smoker, former smoker (cessation of smoking >6 months) and current smoker (daily smoking >6 moths) [27]. Current smokers were further divided into two groups (<20 cigarettes/day and ≥20 cigarettes/day) [28]. Physical activities were measured by weekly total activities; men with regular exercise ≥2 h/week were considered physically active [29]. Alcoholic drinkers were defined as those who had ever consumed three or more drinks (beer, wine, and hard liquor) weekly and done so for six consecutive months [30].

**Figure 3:**
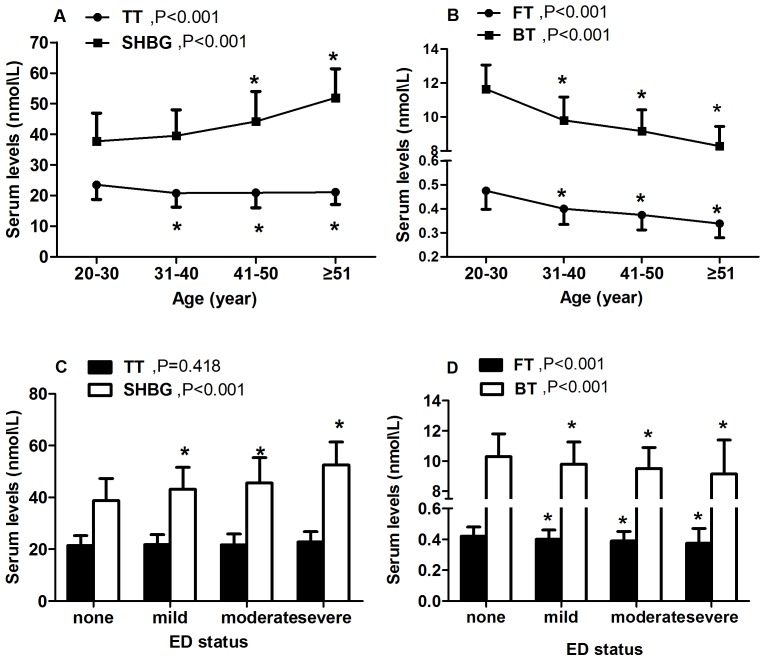
Testosterone and SHBG levels across ages and ED status. Panels A and B. Changes of TT, SHBG, FT, BT by each decade of age; Panels C and D. Association of TT, SHBG, FT, BT with ED status. P value is obtained by one-way analysis of variance (ANOVA). Student-Newman-Keuls test is used for multiple comparisons; asterisk in panels A and B stands for P<0.05 compared with the 20–30 yr age group, whereas in panels C and D stands for P<0.05 compared with the none-ED group. Dots represent median values of sex hormones, while bars represent the 25–75th percentiles. Abbreviations: ED, erectile dysfunction; SHBG, sex hormone-binding globulin; TT, total testosterone; FT, free testosterone; BT, bioavailable testosterone. Testosterone value.

**Table 2:** Association between testosterone and ED in the multivariate regression analysis †.

	IIEF-5 scores					
	Unadjusted		Age-adjusted		Multivariate adjusted †	
TT	−0.03	(−0.51, 0.00); P = 0.065	−0.05	(−0.08, −0.03); P<0.001	−0.04	(−0.07, −0.01); P = 0.006
FT	3.81	(2.29, 5.34); P<0.001	−0.88	(−2.48, 0.72); P = 0.283	−0.78	(−2.38, 0.83); P = 0.344
BT	0.16	(0.09, 0.22); P<0.001	−0.04	(−0.10, 0.03); P = 0.276	−0.03	(−0.10, 0.03); P = 0.347
	**Erectile dysfunction**					
	**Unadjusted**		**Age-adjusted**		**Multivariate adjusted** †	
TT	1.01	(1.00, 1.03); P = 0.151	1.02	(1.01, 1.04); P = 0.002	1.02	(1.00, 1.04); P = 0.021
FT	0.14	(0.06, 0.33); P<0.001	1.05	(0.41, 2.68); P = 0.921	0.98	(0.38, 2.55); P = 0.988
BT	0.92	(0.89, 0.96); P<0.001	1	(0.96, 1.04); P = 0.903	1	(0.96, 1.04); P = 0.992

† Multivariate adjusted for age, smoking status, alcoholic drinking, hypertriglyceridemia, hyperglycemia, elevated BP, low HDL-C and central obesity. Linear regression is conducted using the IIEF-5 score as a continuous variable, whereas the binary logistic regression is conducted using ED defined as a dichotomous variable using a cut-off point of IIEF-5<22. ED = erectile dysfunction; IIEF-5 = 5-item International Index of Erectile Function; TT = total testosterone; FT = free testosterone; BT = bioavailable testosterone; BP = blood pressure, HDL-C = high-density lipoprotein cholesterol.

**Table 3:** Subgroup analysis of association between ED and FT, BT in the univariate binary logistic models.

	Odd Ration (95% Confidence Interval)			
	FT	P	BT	P
Cigarette smoking				
Never smoker	0.08 (0.02–0.33)	<0.001	0.90 (0.86–0.96)	<0.001
<20 cigarettes per day	0.62 (0.13–2.93)	0.548	0.98 (0.92–1.05)	0.548
≥20 cigarettes per day	0.06 (0.01–0.33)	0.001	0.89 (0.83–0.96)	0.001
Alcoholic drinking				
Never drinker	0.07 (0.01–0.85)	0.037	0.90 (0.81–0.99)	0.041
<3 drinks per week	0.19 (0.07–0.52)	0.001	0.93 (0.90–0.97)	0.001
≥3 drinks per week	0.08(0.01–0.77)	0.029	0.90 (0.82–0.99)	0.031
Physical activities				
<2 hours per week	0.14(0.05–0.38)	<0.001	0.92(0.89–0.96)	<0.001
≥2 hours per week	0.14 (0.03–0.78)	0.024	0.92(0.86–0.99)	0.025
Metabolic Syndrome				
No	0.17 (0.07–0.42)	<0.001	0.93 (0.90–0.97)	<0.001
Yes	0.03 (0.00–0.78)	0.035	0.87 (0.76–0.99)	0.035
Hypertriglyceridemia				
No	0.15 (0.06–0.42)	<0.001	0.93 (0.89–0.97)	<0.001
Yes	0.08 (0.01–0.49)	0.001	0.90 (0.84–0.97)	0.001
Hyperglycemia				
No	0.21 (0.08–0.57)	<0.001	0.94 (0.90–0.98)	0.003
Yes	0.10 (0.02–0.58)	0.01	0.91 (0.85–0.98)	0.011
Elevated BP				
No	0.15 (0.06–0.40)	<0.001	0.93 (0.89–0.96)	<0.001
Yes	0.15 (0.02–1.22)	0.076	0.93 (0.85–1.01)	0.076
Low HDL-C				
No	0.13 (0.05–0.31)	<0.001	0.92 (0.89–0.95)	<0.001
Yes	0.71(0.01–40.7)	0.869	0.98 (0.83–1.16)	0.835
Central obesity				
No	0.12 (0.04–0.31)	<0.001	0.92 (0.88–0.95)	<0.001
Yes	0.21 (0.03–1.66)	0.139	0.94 (0.86–1.02)	0.14

TT = total testosterone, FT = free testosterone, BT = bioavailable testosterone, SHBG = sex hormone-binding globulin, BP = blood pressure, HDL-C = high-density lipoprotein cholesterol.

### Serum Assay

The description of the laboratory test has been previously reported in detail [21], [22]. Briefly, about 10 ml overnight fasting venous blood specimens were collected between 8∶00 and 11∶00 in the morning and were transported frozen to the testing center of Department of Clinical Laboratory at the First Affiliated Hospital of Guangxi Medical University in Nanning in two hours, which were centrifuged within 15 to 25 minutes and stored at −80C until analysis. Triglycerides, HDL-c, and serum glucose were measured enzymatically on a Dimension-RxL Chemistry Analyzer (Dade Behring, Newark, DE) in the Department of Clinical Laboratory at the Fangchenggang First People’s Hospital. Serum TT and sex hormone binding globulin (SHBG) were measured with electrochemiluminescence immunoassay on COBAS 6000 system E601(Elecsys module) immunoassay analyzer (Roche Diagnostics, GmbH, Mannheim, Germany) with the same batch of reagents, and the inter-assay coefficient of variation was 3.6% and 4.4%, respectively. BT and FT were calculated from a validated formula based on equilibrium-binding theory suggesting good agreement with laboratory assay [31], [32].

### Statistical Analysis

All statistical analyses were performed using SPSS version 18.0 software (SPSS Inc., Chicago, IL, USA). The continuous variables were examined by Shapiro-Wilks test. Sex hormones including TT, FT, BT and SHBG, not conforming to a normal distribution, were logarithmically transformed in the following analysis, whereas the back-transformed values were reported. Categorical variables were presented with frequencies and proportions. Baseline characteristics were compared between cases (ED) and controls (non-ED) with Mann–Whitney u-test and χ2 test where appropriate. The sample was divided into four groups according to each decade of age: 20–30 (N = 540), 31–40 (N = 682), 41–50 (N = 346), ≥51–60 (N = 198). ED status was classified into four categories as none (N = 931), mild (N = 621), moderate (N = 169), and severe (N = 55). Changes of testosterone and SHBG levels across the four age groups or ED status were evaluated by one-way analysis of variance (ANOVA), thus Student-Newman-Keuls test was used for multiple comparisons. Correlations of age with sex hormones including TT, FT, BT and SHBG were examined via Spearman correlation analyses. When IIEF-5 score was used as a continuous variable to indicate the increased severity of ED, the linear regression models were used to assess the association between testosterone and IIEF-5. Analyses were repeated in the binary logistic regression models when ED was defined as a dichotomous variable using a cut-off point of IIEF-5<22. Both the linear regression and the binary logistic regression were constructed in unadjusted, age-adjusted and multivariate adjusted models. The multivariate adjusted model included the following covariates: age, smoking status, alcoholic drinking, physical activity, hypertriglyceridemia, hyperglycemia, elevated BP, low HDL-C and central obesity. Statistical tests were two-tailed, and a P value<0.05 was considered statistically significant.

## Results

### General Characteristics of the Studied Sample

Characteristics of the 1,776 men in our study were presented in Table 1. There were 47.6% of men with ED (IIEF-5<22). Between the ages of 20 and 77, the prevalence of mild ED increased from 28.7% to 42.3%, moderate ED increased from 6.5% to 24.2%, and severe ED increased from 1.7% to 10.1%. As showed in Figure 2, prevalence of ED increased with age.

The median age of ED group was 40 (Table 1). Men in ED group were significantly older than men in non-ED group (P<0.001). The proportion of men with smoking or drinking showed very little differences between ED group and non-ED group (P = 0.050, P = 0.018). The proportion of men with regular exercise ≥2 h/week did not show significant difference between ED group and non-ED group (P = 0.922). Regarding the components of metabolic syndrome, men with ED were more prone to have elevated BP (P = 0.036) and hyperglycemia (P<0.001).

Both FT and BT were inversely correlated with age (both r = −0.482, P<0.001). Although TT was inversely correlated with age as well (r = −0.169, P<0.001), its reduction across the age groups was relatively small in absolute terms (Figure 3). Both FT and BT gradually increased with advancing age (P<0.001), parallel to its decrease with increasing severity of ED (P<0.0 01). Similarly, SHBG gradually increased with advancing age (P<0.001), parallel to its increase with increasing severity of ED (P<0.0 01). However, the significant decrease of TT across the age (P<0.0 01) groups was not parallel with its obscure increase across the ED status (P = 0.418).

### Association between Testosterone and ED

Table 2 presented the unadjusted, age-adjusted, and multivariate adjusted association between TT, FT, BT and ED. TT was inversely associated with IIEF-5 after adjusting for age [β = −0.05; 95% confidence interval (CI): −0.08, −0.03], and it was associated with a risk of ED in the age-adjusted model [odds ratio (OR)  = 1.02, 95% CI: 1.00–1.04]. After adjusting the following variables including smoking status, alcoholic drinking, physical activity, hypertriglyceridemia, hyperglycemia, elevated BP, low HDL-C and central obesity, the positive association between ED and TT remained significant.

FT and BT were positively associated with IIEF-5 scores (β = 3.81 and 95%CI: 2.29–5.34, β = 0.16 and 95%CI: 0.09–0.22, respectively), however, the positive associations were not statistically significant after adjusting for age (Table 3). In the unadjusted model of binary logistic regression analysis, FT was associated with ED with an OR of 0.14 (95%CI: 0.06–0.33), and BT was associated with ED with an OR of 0.92 (95%CI: 0.89–0.96). The inverse associations between FT, BT and ED were still observed in subgroup analysis (Table 3); however, in men with elevated BP, low HDL-C or central obesity, the association was detected without statistical significance.

## Discussion

The present study in a large series of Chinese men from general population reveals that serum levels of FT and BT are decreased with age, whereas TT do not change much with age presumably because the SHBG increases as well. FT and BT are inversely related to worsening ED, whereas the positive association between TT and ED is most likely due to the increase in SHBG.

The ED prevalence of our studied sample was 47.6%, closed to the prevalence of 49.4% in the primary care setting [4], 52% in Massachusetts Male Aging Study (MMAS) [33] and 47% in BACH Survey [25]. The IIEF-5 instrument was previously used in China [34], and it was reported that among Chinese men above 40 the prevalence of ED was 40.2% [3]. Additionally, the BACH survey has demonstrated the contribution of modifiable lifestyle factors (physical activity, smoking, and alcohol consumption) to the prevalence of ED [25]. Consistent with the results from BACH [28], we have reported that heavy smokers (≥20 cigarettes/day) had a significantly increased risk of ED than never smokers [23], which had some implications in the present study. Moreover, previous studies have reported that current smokers, alcoholic drinkers had a higher level of FT, and physical activity was positively associated with FT [27], however, in the subgroup analysis of our study, the effect of these lifestyles did not attenuate the inverse association between ED and FT or BT. Similarly, the inverse association between ED and FT or BT appeared to be independent of hypertriglyceridemia and hyperglycemia, whereas in groups with elevated BP, low HDL-C or central obesity, it might be underpowered to detect the relatively small associations observed in other groups.

Concerning the changes of testosterone levels across age, FT or BT rather than TT appeared to be more correlated with age [9]. We explained that TT did not change much with age probably because SHBG went up with age [9], [18]. It has been reported that serum testosterone levels gradually fall with advancing age, whereas SHBG levels increase with age and present a rapid increase in the old [35], consistent with the current study. Along these lines, although FT and BT were decreased among the aged men, the unbound testosterone may have a high probability to combine with SHBG because the elevation of SHBG level showed an enhanced rate when these men got older. Thus the net result of these changes of unbound testosterone and SHBG is to present that TT did not change much with age. Moreover, TT levels in many aged men were very closed to those found in young healthy subjects [36], which might indirectly support this result from present study. Additionally, we observed that the increase of SHBG across ED status paralleled with its increase with age, thus the positive association of SHBG with ED might be a reflection of age. However, we observed that the association of TT with ED and its association with age showed a reverse direction, thus the positive association between TT and ED was probably due to the increase in SHBG.

It has been widely accepted that circulating testosterone is partly bound to SHBG with high affinity, so that testosterone levels are strongly related to SHBG concentrations [37]. In addition, genetic variants in the SHBG locus have been associated with a substantial variation in testosterone concentrations, and the SHBG polymorphism could affect testosterone binding to SHBG [38]. Although the association between TT and ED in the current study was independent of age, the possibility of SHBG interaction could not be ruled out. As shown in our study, both TT and SHBG were gradually increased across the ED status, although the increase in TT was relatively small in absolute terms. Moreover, it is critical to highlight that although the positive association between TT and ED remained statistically significant after adjusting the putative confounders, the magnitude of this association was modest.

Recently, the EMAS demonstrated that there was a testosterone threshold for the relationship between TT and ED [5], [39]. TT was associated with worse sexual functioning at concentrations of 8 nmol/l or less, whereas the relationship came to a plateau at TT levels over 8 nmol/l [5]. This finding was consistent with the evidence from the animal trials that androgen requirement for sexual behavior was less than the amount normally present [40], [41] Moreover, evidences from a meta-analysis also suggested that such a testosterone threshold on sexual function might exist in men [42]. Nevertheless, the EMAS group also suggested that the relationship between testosterone and sexual function might be different in older compared with younger men [5]. In this case, our study based on a relatively young population might emerge with some important values. We found FT or BT was inversely related to worsening ED in these patients, and suggested the threshold effect between TT and ED take into account the increase in SHBG.

Although the strength of the present study was characterized by its large sample size and its provenience from general population, some important limitations must be recognized. The cross-sectional nature of the study does not allow identifying the causality but only the associations. The FAMHES population was of southern Chinese Han ethnicity, thus the extrapolation to other ethnic groups should be done with caution. In addition, our studied sample was a very young population (for instance, compared to MMAS). Although IIEF-5 is a validated instrument for ED assessment widely used in both clinical and epidemiologic studies, the definition of ED according to an IIEF-5 score of <22 is blunt when considering Yes versus No statistics. It might result in extremely mild ED being considered a Yes, lumped together with very different and more debilitating ED of score 5. Nevertheless, it has been recently reported that men with mild ED have similar risk factors to a general ED clinical trial population [43]. Although previous study observed a higher prevalence of psychogenic ED in younger patients and organic ED in older patients [15], there were insufficient evidence identifying the putative prevalent component for ED in our study. Although there were no significant differences with respect to the ED prevalence between subjects who participated in the study and those who did not, not all the men responding to the questionnaire had voluntarily measured their sex hormones levels, so that selection bias might still exist because our response rate of hormones measurement was 67.6%. Although the mass spectrometry-based methods [e.g. gas chromatography-mass spectrometry (GC-MS)] with improved accuracy and precision in serum testosterone measurements has been recommended in studies of male sexual function [44], serum testosterone in the present study was measured with electrochemiluminescence immunoassay [31], [32]. TT levels were quite high in our population, and we have limited information on the causes of these unexpectedly high TT levels, which were probably due to other sexual behaviors rather than ED [42], such as increased frequency of autoeroticism or masturbation [45], and high prevalence of extramarital affairs [46].

This cross-sectional study was conducted in a large series of Chinese men from general population. Serum levels of FT and BT were decreased with age, whereas TT did not change much with age presumably due to the increase in SHBG. BT and FT were inversely related to worsening ED. The positive association between TT and ED is most likely due to the increase in SHBG, thus the threshold effect between TT and ED needs further investigation.
